# Prescribing Errors and Pharmacist Interventions in Paediatric Primary Health Care in Saudi Arabia: A Mixed-Methods Study

**DOI:** 10.3390/healthcare14060810

**Published:** 2026-03-22

**Authors:** Anwar A. Alghamdi, Wael Y. Khawagi, Abdullah A. Alshehri, Roaa I. Saif, Bayan A. Alasmari, Esraa M. Binjabi, Fawwaz M. Alamri, Aftab Ahmad

**Affiliations:** 1 Health Information Technology Department, The Applied College, King Abdulaziz University, Jeddah 21589, Saudi Arabia; fmaalamri@kau.edu.sa (F.M.A.); abdulsalam@kau.edu.sa (A.A.); 2 Pharmacovigilance and Medication Safety Unit, Center of Research Excellence for Drug Research and Pharmaceutical Industries, King Abdulaziz University, Jeddah 21589, Saudi Arabia; 3 Department of Clinical Pharmacy, College of Pharmacy, Taif University, Taif 21944, Saudi Arabia; w.khawagi@tu.edu.sa (W.Y.K.); a.aalshehri@tu.edu.sa (A.A.A.); 4 Pharmaceutical Services Department, Medical Services Center, King Abdulaziz University, Jeddah 21589, Saudi Arabia; rasaif@kau.edu.sa (R.I.S.); baalasmari@kau.edu.sa (B.A.A.); ibinjabi@kau.edu.sa (E.M.B.)

**Keywords:** paediatric prescribing errors, primary health care, pharmacist interventions, medication safety, weight-based dosing, mixed-methods study

## Abstract

**Background:** Medication use in paediatric populations is inherently complex and carries a heightened risk of prescribing errors, particularly within primary health-care settings. Despite this concern, evidence describing paediatric prescribing errors in Saudi Arabia remains scarce. Hence, the present study aimed to evaluate the prevalence and patterns of prescribing errors in paediatric primary care and to characterize the pharmacist-led interventions undertaken to resolve these errors. **Methods:** A prospective, mixed-methods cross-sectional study was conducted over three months at a primary health-care centre. Paediatric outpatient prescriptions were systematically reviewed during routine practice by trained clinical pharmacists. All suspected errors were independently validated and classified for severity by a multidisciplinary expert panel. Descriptive statistics were used to summarise prescribing errors, and associations with patient and prescription characteristics were assessed using chi-square tests. Qualitative data were analysed using a descriptive thematic approach to explore mechanisms of error identification and the nature of corrective pharmacist interventions. **Results:** A total of 545 paediatric outpatient prescriptions were reviewed, of which 142 prescriptions (26.1%) contained at least one prescribing error. Across these prescriptions, a total of 145 individual prescribing errors were identified. Dose-related errors were the most common (68.3%), followed by inaccuracies in dosing frequency (11.0%) and inappropriate drug selection (9.0%). The occurrence of prescribing errors was significantly associated with patient weight (*p* = 0.016), the number of medications per prescription (*p* < 0.001), and the recorded diagnosis (*p* = 0.018). The majority of errors were intercepted prior to medication dispensing (93.0%), and no cases of patient harm were identified. Qualitative analysis revealed that errors were predominantly detected through cross-checking with authoritative drug references, recalculation of weight-based doses, and application of clinical judgement, and were most often resolved through direct communication with the prescribing clinician. **Conclusions:** Prescribing errors occur frequently in paediatric outpatient settings; however, most are preventable with appropriate safeguards. Pharmacists play a critical role in identifying and resolving these errors before they result in patient harm. Enhancing paediatric prescribing support systems and strengthening interprofessional collaboration may further advance medication safety within primary health-care services.

## 1. Introduction

Medication use in paediatric populations is inherently complex and is associated with a greater risk of prescribing errors than in adults [1,2]. Accurate paediatric dosing frequently requires individualized calculations that account for age, body weight, body surface area, and developmental maturity. Growth velocity also varies throughout childhood; therefore, regular verification of a child’s current body weight is essential to ensure accurate weight-based dosing [3]. In addition, many medicines are prescribed off-label in children because of the limited availability of paediatric formulations and the scarcity of robust clinical trial evidence [4,5,6]. When these factors are combined with time constraints and high workload in outpatient care settings, the probability of prescribing errors increases, potentially undermining therapeutic effectiveness and patient safety. Prescribing errors constitute a substantial proportion of medication errors and are widely recognised as preventable events that occur during the processes of drug selection and prescription writing [7,8,9]. In paediatric practice, common prescribing errors include incorrect dose calculations, inappropriate dosing frequency or duration, selection of unsuitable medicines for a child’s age, and omission of essential prescription details [10,11]. Dose-related errors are particularly concerning, as even small deviations from recommended ranges can result in subtherapeutic treatment or toxicity, especially in infants and young children with limited physiological reserves [2,4].

International studies have reported wide variations in the prevalence of paediatric prescribing errors across health-care settings, with outpatient and primary care environments receiving comparatively less attention than hospital inpatient wards [12,13,14]. However, primary health-care centres manage a large volume of paediatric encounters and prescriptions, making them a critical setting for medication safety research [15,16]. In many low- and middle-income and rapidly developing health systems, data on paediatric prescribing quality in ambulatory care remain scarce, limiting the ability to design targeted safety interventions. Pharmacists play a pivotal role in the identification, interception, and resolution of prescribing errors before medications reach the patient.

Through systematic prescription review, reference checking, and clinical judgement, pharmacists act as a final safeguard within the medication-use process [9,17,18]. Beyond quantifying error rates, understanding how errors are detected and resolved in real-world practice provides valuable insight into the mechanisms of error prevention and interprofessional collaboration [2,19]. In Saudi Arabia and similar health-care contexts, evidence on paediatric prescribing errors in primary care is limited, particularly studies combining quantitative assessment of error prevalence with qualitative exploration of detection and corrective processes. Addressing this gap is essential to inform policy, training, and system-level improvements aimed at enhancing paediatric medication safety. Therefore, the current study aimed to determine the prevalence and characteristics of prescribing errors in paediatric outpatient prescriptions at a university-affiliated primary health-care centre, examine associations between prescribing errors and patient- and prescription-related factors, and qualitatively explore the methods used by pharmacists to detect prescribing errors and the corrective actions undertaken following their identification.

## 2. Methods

### 2.1. Study Design and Setting

An observational study with a prospective cross-sectional design was conducted over a three-month period from March to May 2025 at a university-affiliated primary health-care center.

The centre serves a large catchment population and receives approximately 300 paediatric visits daily. Prescriptions issued during routine outpatient encounters formed the basis of this investigation.

### 2.2. Study Population

All outpatient prescriptions issued to pediatric patients aged <18 years during the study period were eligible for inclusion. Prescriptions generated during routine clinical encounters for either acute or chronic medical conditions were included. Prescriptions issued to patients aged ≥18 years were excluded from the analysis.

### 2.3. Prescribing Errors Detection

Before data collection, all participating pharmacists completed structured training on the study protocol, definitions of prescribing errors, and the data-entry procedures. Trained clinical pharmacists prospectively reviewed paediatric prescriptions as part of their routine clinical duties. Potential errors were identified primarily through clinical judgement, supported by standard paediatric references and predefined error-classification criteria.

Prescribing errors were defined in accordance with the National Coordinating Council for Medication Error Reporting and Prevention (NCC MERP) as “preventable events that may result in inappropriate medication use or patient harm at any stage while the medication remains under the control of a health-care professional, patient, or consumer” [20]. Each prescription was evaluated against established pediatric reference sources, including the British National Formulary for Children (BNFc), the Lexicomp^®^ Pediatric & Neonatal Dosage Handbook, and relevant Saudi Ministry of Health (MOH) clinical guidelines, where applicable [21,22].

Operationally, a prescribing error was defined as a deviation from evidence-based recommendations related to drug selection, dose, frequency, duration, route of administration, dosage form, or clinically significant drug–drug interactions. Dose errors were identified when prescribed doses fell outside the recommended weight- or age-based ranges according to BNFc or Lexicomp guidance, allowing for a clinically acceptable margin unless the drug had a narrow therapeutic index. Off-label prescribing was not automatically classified as an error. Off-label use supported by recognized pediatric references (e.g., BNFc or Lexicomp) was considered acceptable clinical practice and was classified as a prescribing error only when it was unsupported by authoritative references or associated with a potential safety concern. All suspected errors were documented and independently validated by a multidisciplinary expert panel.

### 2.4. Data Collection

Data were collected prospectively from outpatient paediatric prescriptions using a standardised data-collection form (Supplementary Materials). The form captured core patient and prescription characteristics, including age, sex, weight, diagnosis, and the total number of medications prescribed. For prescriptions in which a potential prescribing error was identified, additional drug-specific information was collected, including the medication name, prescribed dose, dosing frequency, duration of therapy, route of administration, and formulation. The data collection tool was developed and content validated by a medication safety expert to ensure clarity, completeness, and consistency. In addition to the structured data fields, pharmacists provided free-text narrative descriptions for each detected error. These narratives detailed how the error was identified, the reference sources used, and the corrective actions implemented. These narrative accounts formed the basis of the qualitative component of the study.

### 2.5. Validation and Severity Assessment

Each suspected prescribing error was independently reviewed by a multidisciplinary expert panel comprising two clinical pharmacists, one pediatric physician, and one medication safety specialist. Errors were confirmed and classified according to the severity categories of the National Coordinating Council for Medication Error Reporting and Prevention (NCC MERP) Index [23]. Initial independent assessments were compared, and any disagreements in classification or severity grading were discussed until consensus was reached. Disagreements were infrequent and typically related to severity categorization rather than the presence of an error. All final classifications were agreed upon by consensus prior to inclusion in the analysis.

### 2.6. Quantitative Analysis

Prescription characteristics were summarised using descriptive statistical methods and the frequency of prescribing errors. Age and weight were grouped according to the WHO developmental stages and standard paediatric weight bands [24]. Diagnosis categories were coded using an ICD-10–based classification system created through predefined rules and text-pattern matching [25]. The primary unit of analysis for prevalence and risk-factor comparisons was the individual prescription, which was classified dichotomously as either containing at least one prescribing error or no prescribing error. For descriptive analyses, each confirmed prescribing error was treated as a separate event.

Associations between prescribing errors and categorical variables, including age, weight, number of medications, and diagnosis, were evaluated by Pearson’s chi-square test. The statistical significance was considered as *p* < 0.05.

To examine independent associations, a multivariable logistic regression model was constructed with prescription-level error status (yes/no) as the dependent variable. Independent variables were selected a priori based on clinical plausibility and prior literature and included age group, weight category, number of medications, and diagnosis group. To enhance model stability and minimize sparse-cell bias, categories were collapsed according to clinical similarity and frequency distribution. Weight was retained in the regression analysis as a separate “missing” category to preserve sample size and to assess whether missingness was associated with prescribing errors. Prescriptions with missing diagnosis information were excluded from the regression analysis due to complete separation (i.e., no observed errors in this category). Adjusted odds ratios (aORs) with 95% confidence intervals (CIs) were reported. All statistical analyses were conducted using Stata version 16 (StataCorp LLC, College Station, TX, USA).

### 2.7. Qualitative Analysis

A qualitative descriptive approach was used to analyse pharmacists’ free-text reports documenting the identification and resolution of prescribing errors. Two reviewers independently conducted line-by-line coding using an inductively developed codebook. The codebook was refined iteratively through discussion to ensure consistent application. Discrepancies were resolved through consensus meetings, and final theme classifications were agreed upon unanimously. Representative quotations were selected to illustrate and support each theme.

### 2.8. Ethical Consideration

Each eligible patient was assigned a unique identification code to ensure confidentiality. A master patient-link code sheet was securely stored on site in a locked cabinet and was not removed from the health-care centre. The final dataset used for analysis contained no identifiable patient information. Ethical approval for this study was waived by the institutional Research Ethics Committee (REC) at King Abdulaziz University (Reference No for exemption (41-25)).

## 3. Results

### 3.1. Prescriptions Characteristics

A total of 545 paediatric prescriptions were evaluated. Most were issued for young children aged 2–6 years (36.8%) and children aged 6–12 years (36.3%), followed by infants aged 1 month–2 years (18.2%). Weight data were available for 433 out of 545 children (79.4%). Nearly half of the patients (49.7%) weighed between 10 and <20 kg. Patients received between one and seven medications per encounter. Most prescriptions contained two medications (26.4%), followed by three medications (25.7%) and four medications (20.6%). Respiratory diseases were the most frequent diagnoses (40.9%). Other common categories included skin and subcutaneous tissue disorders (10.8%), health-service encounters such as vaccinations or routine examinations (9.5%), endocrine and metabolic conditions (7.2%), and digestive system disorders (7.0%). Prescription characteristics are summarised in Table 1.

### 3.2. Distribution of Prescribing Errors Across Patient and Prescription Characteristics

The distribution of prescriptions with and without errors across demographic and clinical categories is presented in Table 1. Overall, prescribing errors occurred in 26.1% of the prescriptions (n = 142). The proportion of errors did not significantly differ by age group (*p* = 0.734), with error rates ranging from 18.3% in infants to 31.1% among adolescents. In contrast, weight category showed a statistically significant association with prescribing errors (*p* = 0.016). Error frequency increased progressively with higher weight groups, from 24.6% in children < 10 kg to 44.7% among those ≥40 kg. A statistically significant relationship was found between the number of prescribed medications and occurrence of prescribing errors (*p* < 0.001).

Prescriptions containing four medications (47.3%) and five or more medications (44.6%) had substantially higher error rates compared to those with one or two medications (11.8% and 11.1%, respectively). Prescribing errors also varied by diagnosis category (*p* = 0.018). Higher error proportions were observed among prescriptions for digestive system disorders (36.8%) and other diagnoses (32.8%), compared with lower rates in health-service encounters (17.3%) and general symptoms or abnormal findings (15.4%). No errors were reported in prescriptions with missing diagnoses.

### 3.3. Prescribing Error Characteristics

Prescribing errors were identified in 142 prescriptions, yielding a prevalence of 26.1%. Among the 142 identified prescribing errors, the most frequently involved drug classes were antihistamines (24.7%), followed by cephalosporins (14.8%), and “non-steroidal anti-inflammatory drugs” (NSAIDs) (11.3%). Other drug classes frequently associated with errors included vitamins (9.9%), antifungal agents (6.3%), non-opioid analgesics (6.3%), and corticosteroids (6.3%). Less commonly affected drug classes were topical corticosteroids combined with antimicrobials (7.8%), leukotriene receptor antagonists (2.8%), antibacterials (4.9%), and anthelmintics, 5HT3-receptor antagonists, and laxatives (each 1.4%). Topical nasal decongestants were the least frequent (0.7%).

The majority of prescribing errors involved oral formulations (78.2%), followed by topical preparations (12.7%) and nasal medications (4.9%). Errors involving other routes of administration were less frequent, including ophthalmic (2.1%), rectal (1.4%), and otic (0.7%) formulations. Following consolidation of overlapping categories, dose-related errors were the most frequent, accounting for 99 cases (68.3%). Errors related to frequency were identified in 16 cases (11.0%), while wrong drug errors occurred in 13 cases (9.0%). Duration-related errors accounted for 9 cases (6.2%), and wrong dosage form errors were the least frequent (8 cases, 5.5%). Overall, a total of 145 individual prescribing errors were confirmed. Three prescriptions contained more than one prescribing error. Most prescribing errors were categorized as Category B (n = 132; 92.96%), indicating that the error occurred but was intercepted before reaching the patient. One error (0.7%) fell under Category C, where the error reached the patient without causing harm, whereas nine errors (6.3%) were categorised as Category D, signifying that the error reached the patient and needed monitoring or clinical intervention to prevent harm. No errors were associated with actual patient harm (Categories E–I). The characteristics of the identified prescribing errors are summarised in Table 2.

Stratification of prescribing error subtypes by age group showed significant variation in distribution (χ^2^ = 66.13, *p* < 0.001). Dose-related errors accounted for the majority of errors in infants (57.7%), young children (69.1%), and children aged 6–12 years (83.0%). In contrast, wrong dosage-form errors were more frequently observed among adolescents (6 of 14 errors).

Stratification of prescribing error subtypes by age group demonstrated a significant variation in distribution (*p* < 0.001) (Table 3). Dose-related errors were the most frequent subtype across all age groups except adolescents, accounting for 57.1% of errors among infants, 69.6% among young children, and 83.0% among children aged 6–12 years. Among adolescents, wrong dosage-form errors constituted the largest proportion (42.9%). Frequency-related errors were more common among young children (16.1%), whereas wrong-drug (17.9%) and wrong-duration (17.9%) errors were proportionally more frequent among infants.

### 3.4. Multivariable Analysis of Factors Associated with Prescribing Errors

In the multivariable logistic regression analysis (n = 513), the number of prescribed medications was the strongest independent factor associated with prescribing errors. Compared with prescriptions containing 1–2 medications, those containing three medications had more than a threefold higher odds of error (aOR 3.21; 95% CI 1.77–5.81; *p* < 0.001), whereas prescriptions containing ≥4 medications had nearly a ninefold higher odds (aOR 8.90; 95% CI 5.00–15.86; *p* < 0.001). Prescriptions issued to children weighing ≥40 kg were also independently associated with increased odds of error (aOR 2.58; 95% CI 1.12–5.94; *p* = 0.026). In addition, endocrine and digestive system diagnoses were independently associated with higher odds of prescribing errors compared with respiratory diagnoses (aOR 2.64; 95% CI 1.34–5.21; *p* = 0.005). Age group was not independently associated with prescribing errors after adjustment for other variables. The results of the multivariable logistic regression analysis are presented in Table 4.

### 3.5. Detection Methods Used in Identifying Prescribing Errors:

A qualitative review of the pharmacists’ comments and documentation revealed several recurring themes that described how medication errors were identified (Table 5). Detection relied primarily on systematic verification against standard references, dose calculation, and clinical judgment.

1. Verification Against Authoritative References:

Most errors were detected by cross-checking prescriptions with trusted sources such as the British National Formulary (BNF), British National Formulary for Children (BNFC), Micromedex, Medscape, Lexicomp, and manufacturer leaflets. Pharmacists verified the appropriateness of dose, frequency, and duration for each prescribed drug. In many cases, deviations from reference values prompted identification of subtherapeutic or excessive doses. “According to BNFC, the reference dose should be 7.5 mg/kg every 8 h; 8 mL is subtherapeutic.”

2. Dose-Related Error Detection:

The most frequently described approach involved recalculating the dose based on the child’s weight. Errors were detected when the prescribed amount exceeded or fell below the reference range. “*The dose was calculated according to the patient’s body weight; it was found to be an overdose*.”

3. Identification of Incorrect Frequency or Duration:

Several comments referred to mismatched dosing intervals or treatment periods compared with guideline recommendations. “*According to BNFC, treatment should continue for at least seven days; the prescription was for five days*.”

4. Contraindications and Age-Related Inappropriateness:

Many errors involved the use of medicines not recommended for specific age groups, as indicated in drug monographs or leaflets. “*Ketoconazole is not recommended for children younger than 12 years*.”

5. Missing or Ambiguous Prescription Information:

Some errors were detected because essential details—such as dose or duration—were omitted or unclear. “*The doctor did not write the dose*.”

6. Post-Dispensing Discovery and Caregiver Feedback:

A few cases were identified retrospectively after the medicine had been dispensed, usually when caregivers returned or reported unexpected dosing instructions. “*After one month of dispensing, I discovered it was an overdose.*”

### 3.6. Corrective Actions Taken Following Error Detection:

Pharmacists documented various actions undertaken to resolve the detected medication errors. The qualitative analysis revealed that corrective measures predominantly involved direct communication between pharmacists and prescribers, usually resulting in an immediate modification of the prescription (Table 6).

1. Collaboration and Communication with Prescribers

In most cases, pharmacists contacted the treating physician by phone or in person to clarify or correct the prescription. Most physicians responded positively and agreed to adjust the medication according to standard references such as the BNF or BNFC. The corrections typically involved dose adjustments, frequency modification, changes in dosage form, or correction of treatment duration. “*The doctor accepted my recommendation to change the frequency to twice daily*.”

2. Dose and Regimen Adjustments:

Most interventions related to dose corrections (increasing, decreasing, or clarifying the prescribed dose) and frequency or duration adjustments to align with reference standards. “*Pharmacist contacted the doctor, and corrected the frequency and duration to seven days*.”

3. Refusal to Dispense Until Correction:

When errors had potential safety implications, pharmacists occasionally refused to dispense the medication until the prescriber reviewed and corrected the prescription. “*Pharmacist refused to dispense until the doctor checked the dose*.”

4. Physician Disagreement or Escalation:

When prescribers declined to modify the prescription or when clarification remained unresolved, pharmacists escalated the case by consulting another physician or referring the patient for further evaluation. “*The doctor refused to change, so Pharmacist consulted another doctor who referred the patient*.”

## 4. Discussion

This study identified prescribing errors in more than one quarter of paediatric outpatient prescriptions in a primary health-care centre, highlighting that medication safety challenges remain prevalent in ambulatory paediatric care. Dose-related errors accounted for the majority of identified errors, underscoring the complexity of weight-based dosing and individualised therapy in children. Prescribing errors were significantly associated with patient weight, number of prescribed medications, and diagnostic category, while age group alone was not a significant determinant. Importantly, most errors were intercepted before reaching the patient, with pharmacists playing a central role in detecting and correcting errors through systematic prescription review and interprofessional communication.

The qualitative findings further elucidated the mechanisms by which errors were identified, including verification against authoritative references, independent dose recalculation, and clinical judgement, as well as the corrective actions undertaken following detection. The prevalence of prescribing errors identified in the current investigation is consistent with rates reported in international research conducted in paediatric outpatient and primary care settings; however, direct comparisons are constrained by methodological heterogeneity and differences in error definitions [12,13,14].

Similar to previous research, dose-related errors emerged as the most common type of prescribing error, reflecting the inherent challenges of paediatric dosing and the frequent need for off-label prescribing [11,26,27,28]. Nevertheless, the error rate identified in this study confirms that primary health-care centres, despite often being perceived as lower-risk environments, represent a critical point in the medication-use process where preventable errors frequently occur [29,30]. Given the high volume of paediatric encounters in primary care, even modest error rates may translate into a considerable absolute burden of unsafe prescribing [14,15,16]. The strong association between polypharmacy and prescribing errors is consistent with existing literature demonstrating that increasing medication burden is a key risk factor for prescribing inaccuracies [31,32]. In contrast to some studies, this study did not find a significant association between age group and error occurrence [33,34]. Instead, higher error rates were observed among heavier children, a finding that has been less frequently explored but may reflect uncertainty at the interface between paediatric and adult dosing practices [35]. This finding may partly reflect the complexity of transitioning from pediatric to adult dosing guidance, as adolescents may continue to exhibit substantial variability in growth and developmental maturity.

Pharmacists play a critical role in detecting prescribing errors through verification of drug selection, recalculation of weight-based doses, and cross-checking with authoritative paediatric drug references. Pharmacists also contribute to patient safety through direct communication with prescribers and implementation of corrective interventions before medication dispensing, thereby preventing potential medication-related harm. The predominance of intercepted errors aligns with previous reports emphasising the critical role of pharmacists as a final safety barrier within the medication-use process [36,37]. The detection methods and corrective actions identified in this study mirror those described in other qualitative investigations, reinforcing the importance of pharmacist-led review and effective communication with prescribers [38,39].

The results of this study have important implications for primary care practice. First, targeted strategies to reduce dose-related errors are essential, including routine documentation of accurate patient weight, consistent use of pediatric dosing references, and structured weight-based dose calculation checks [6]. Second, prescriptions involving multiple medications warrant heightened scrutiny, given their strong association with prescribing errors [32]. The findings also highlight the value of embedding pharmacists more formally within pediatric outpatient care pathways. Strengthening pharmacist–prescriber communication, supported by clear institutional policies that empower pharmacists to intervene when safety concerns arise, may further reduce the risk of errors reaching patients. Furthermore, electronic prescribing systems incorporating pediatric-specific clinical decision support and automated dose calculators may provide a sustainable strategy for enhancing prescribing safety [40].

Future studies should examine paediatric prescribing errors across multiple primary care centres in Saudi Arabia to enhance generalisability and explore variability between institutions. Longitudinal studies assessing the impact of specific interventions, such as electronic decision support, standardised dosing tools, or enhanced pharmacist involvement, on prescribing error rates are also warranted. Further qualitative research exploring prescribers’ perspectives on paediatric dosing challenges may complement pharmacist-focused findings and inform the development of targeted educational interventions. In addition, research examining caregiver understanding and involvement in medication use could provide insight into additional safety barriers beyond the clinical setting [41].

This study has several strengths, including its systematic application of standardised definitions and severity classification, and integration of qualitative data to provide contextual insight into error detection and correction. The use of a multidisciplinary panel for error validation further strengthens the reliability of the results. However, the investigations were carried out at a single centre, which may limit the generalisability of the results. Reliance on pharmacist-detected errors may have led to underestimation of true error prevalence, particularly for errors that were not recognised during routine review. In addition, the study did not directly investigate prescriber-related cognitive or organizational factors that may contribute to the occurrence of prescribing errors. Finally, the study did not assess clinical outcomes beyond error severity classification, limiting conclusions regarding long-term patient impact.

## 5. Conclusions

Prescribing errors occur relatively frequently in paediatric outpatient care but are largely preventable. In this study, dose-related errors were the most common subtype, reflecting the challenges of weight-based dosing and individualized pharmacotherapy in children. Prescriptions involving multiple medications (polypharmacy) were also strongly associated with an increased likelihood of prescribing errors.

Importantly, most errors were identified and intercepted by pharmacists prior to medication dispensing, preventing potential patient harm. These findings highlight the critical role of pharmacists in ensuring medication safety through systematic prescription review, verification of weight-based doses, and communication with prescribers.

Strengthening accurate patient weight documentation, structured dose-calculation checks, and the integration of paediatric-specific clinical decision support within electronic prescribing systems, alongside enhanced interprofessional collaboration, may further improve medication safety in paediatric primary health-care settings.

## Figures and Tables

**Table 1:** Characteristics of paediatric prescriptions evaluated in the study (N = 545).

Category	Without Error (n, %)	With Error (n, %)	*p* Value	Total n (%)
**Age group**				
Neonate (Birth–30 days)	2 (100)	0 (0)	0.734	2 (0.4)
Infant (1 month–2 years)	73 (73.7)	26 (18.3)		99 (18.2)
Young child (2–6 years)	145 (72.5)	55 (38.7)		200 (36.8)
Child (6–12 years)	150 (76.14)	47 (33.1)		197 (36.3)
Adolescent (12–18 years)	31 (68.9)	14 (9.9)		45 (8.3)
Missing	2 (100)	0 (0)		2 (0.4)
**Weight Category**				
<10 kg	43 (75.4)	14 (24.6)	0.016	57 (10.5)
10–<20 kg	159 (74.0)	56 (26.0)		215 (39.4)
20–<30 kg	65 (73.9)	23 (26.1)		88 (16.2)
30–<40 kg	22 (62.9)	13 (37.1)		35 (6.4)
≥40 kg	21 (55.3)	17 (44.7)		38 (7.0)
Missing	93 (83.0)	19 (17.0)		112 (20.6)
**Number of medications**				
1 medication	82 (88.2)	11 (11.8)	0.000	93 (17.1)
2 medications	128 (88.9)	16 (11.1)		144 (26.4)
3 medications	103 (73.6)	37 (26.4)		140 (25.7)
4 medications	59 (52.7)	53 (47.3)		112 (20.6)
5 or more medications	31 (55.4)	25 (44.6)		56 (10.3)
**Diagnosis Category**				
Respiratory diseases	159 (71.3)	64 (28.7)	0.018	223 (40.9)
Skin and subcutaneous tissue disorders	44 (74.6)	15 (25.42)		59 (10.8)
Health services/routine encounters	43 (82.7)	9 (17.3)		52 (9.5)
Endocrine, nutritional and metabolic disorders	30 (76.9)	9 (23.1)		39 (7.2)
Digestive system disorders	24 (63.2)	14 (36.8)		38 (7.0)
Infectious and parasitic diseases (non-resp.)	13 (72.2)	5 (27.8)		18 (3.3)
General symptoms and abnormal findings	11 (84.6)	2 (15.4)		13 (2.4)
Other diagnoses (all categories <10)	49 (67.1)	24 (32.8)		73 (13.4)
Missing	30 (100.00)	0 (0.00)		30 (5.5)

**Table 2:** Summary of prescribing error characteristics across categories (n = 142).

Category	n (%)
**Route of administration**	
Oral	111 (78.2)
Topical	18 (12.7)
Nasal	7 (4.9)
Ophthalmic	3 (2.1)
Rectal	2 (1.4)
Otic	1 (0.7)
**Prescribing Error type**	
Wrong dose	99 (68.3)
Wrong frequency	16 (11.0)
Wrong drug	13 (9.0)
Wrong duration	9 (6.2)
Wrong dosage form	8 (5.5)
**Drug class involved**	
Antihistamines	35 (24.7)
Cephalosporins	21 (14.8)
NSAIDs	16 (11.3)
Vitamins	14 (9.9)
Topical corticosteroid + antimicrobial	11 (7.8)
Antifungal agents	9 (6.3)
Non-opioid analgesics	9 (6.3)
Corticosteroids	9 (6.3)
Antibacterial	7 (4.9)
Leukotriene receptor antagonists	4 (2.8)
Anthelmintics	2 (1.4)
5HT3-receptor antagonists	2 (1.4)
Laxatives	2 (1.4)
Topical nasal decongestants	1 (0.7)
**Severity Category**	
B: An error occurred but was intercepted before reaching the patient.	132 (93.0)
C: The error reached the patient but did not result in harm.	1 (0.7)
D: The error reached the patient and required monitoring or clinical intervention to prevent harm.	9 (6.3)

Note: More than one error type may occur in the same prescribing error.

**Table 3:** Distribution of prescribing error types by age group.

Prescribing Error Type	Infants %	Young Children %	Children %	Adolescents %
Wrong dose	57.1	69.6	83.0	35.7
Wrong frequency	7.1	16.1	6.4	14.3
Wrong drug	17.9	8.9	6.4	0.0
Wrong duration	17.9	5.4	0.0	7.1
Wrong dosage form	0.0	0.0	4.3	42.9

**Table 4:** Multivariable logistic regression analysis of factors associated with prescribing errors.

Variable	Adjusted OR	95% CI	*p* Value
Age group			
≤2 years	Reference		
2–12 years	1.17	0.65–2.11	0.599
≥12 years	1.63	0.61–4.37	0.328
Number of medications			
1–2 meds	Reference		
3 meds	3.21	1.77–5.81	<0.001
≥4 meds	8.90	5.00–15.86	<0.001
Weight category			
<20 kg	Reference		
20–<40 kg	1.11	0.64–1.93	0.719
≥40 kg	2.58	1.12–5.94	0.026
Missing weight	0.74	0.38–1.45	0.379
Diagnosis group			
Respiratory	Reference		
Endocrine/Digestive	2.64	1.34–5.21	0.005
Other diagnoses	1.33	0.82–2.14	0.244

**Table 5:** Summary of Themes Related to Prescribing Error Detection.

Theme	Description
Verification against references	Comparison with BNFC, BNF, Micromedex, leaflets
Dose-related detection	Weight-based recalculation revealing over-/underdose
Wrong frequency/duration	Prescribed schedule inconsistent with references
Age contraindication	Medicine not approved for patient’s age
Missing or unclear information	Incomplete dose or duration details
Post-dispensing discovery	Error noticed after use or through caregiver feedback

**Table 6:** Summary of Corrective Actions Taken Following Detection of Prescribing Errors.

Theme	Description
Communication and agreement with prescriber	Pharmacist contacted the doctor and reached consensus to modify the prescription
Dose or regimen correction	Adjustments in dose, frequency, duration, or dosage form based on references
Refusal to Dispense Until Correction	Pharmacist withheld the medication until correction was made
Physician Disagreement or Escalation	Doctor declined to modify the prescription

## Data Availability

The raw data supporting the findings of this study will be made available by the authors upon reasonable request.

## Supplementary Materials

The following supporting information can be downloaded at: https://www.mdpi.com/article/10.3390/healthcare14060810/s1, Table S1. Master Patient Link Code Sheet.
