# Supplementary material for: Prescribing Errors and Pharmacist Interventions in Paediatric Primary Health Care in Saudi Arabia: A Mixed-Methods Study
Source: Healthcare (Basel). 2026 Mar 22;14(6):810. doi: 10.3390/healthcare14060810 (PMC13026935; doi:10.3390/healthcare14060810)
Supplement: Supplementary file 1 [file healthcare-14-00810-s001.zip › healthcare-4149282-supplementary.pdf]

Data Collection Forms

Table S1. Master Patient Link Code Sheet

(To be securely stored in filing cabinet and not to leave the University Medical Services Centre)

Data collector:			
Date:			
Patient medical number	Patient link code (study number)	Patient medical number	Patient link code (study number)

Data collection form (A): Patient information

Please fill the patient details in the following table	
Patient study number	

(Can be found on the Master Patient Link Code Sheet)			
Date (dd/mm/yy)	…. ./..…/……...	Age	………months or ………years
Number of medications prescribed		Weight	………kg
History of drug allergies or drug intolerances	□ Yes □ No If yes, please list: ………………………… … …………………………… …………………………… ……………………………	Diagnosis	……………………
Data collector			

Data collection form (B): Medication Error (ME) information

Please complete one form for each suspected ME		
**Patient study number**	**Case number** (e.g. 1st or 2nd ME for this patient)	**Date of ME Detection** (dd/mm/yy)
		…. /…/…….
**Stage of suspected ME** *(Please check stage that apply, if more than one select the primary process problem)*		

☐ **Prescribing:**<br>Description:…………………………………………………………………<br>…………………………………………………………………………………<br>…………………………………………………………………………………<br>…	☐ **Preparation:**<br>Description:…………………………………………………………………<br>…………………………………………………………………………………<br>…………………………………………………………………………………<br>…………………………………………………………………………………<br>…
☐ **Dispensing:**<br>Description:…………………………………………………………………<br>…………………………………………………………………………………<br>…………………………………………………………………………………<br>…	☐ **Monitoring:**<br>Description:…………………………………………………………………<br>…………………………………………………………………………………<br>…………………………………………………………………………………<br>…

**Medication involved**	
Name of drug	
Route of administration	
Dose & frequency	
Total doses this patient received	

Please provide a full description of the event:

How was the event detected?

……

……

……

What was the corrective action?

……

Was there any outcome for patient? *(If the patient has already been using the drug, was there any associated **harm**?)*

☐ **No** ☐ **Yes,** please provide description of **harm**: ………………………………………………………………………………

If ***yes***, please rate the severity of the harm:

☐ **E. An error occurred that resulted in the need for treatment or intervention and caused temporary patient harm.**

Description: ……

☐ **F. An error occurred that resulted in initial or prolonged hospitalisation and caused temporary harm.**

Description: ……

☐ **G. An error occurred that resulted in permanent patient harm.**

Description: ……

☐ **H. An error occurred that resulted in near-death event** (e.g. anaphylaxis, cardiac arrest)

Description: ……

☐ **I. An error occurred that resulted in patient death.**

Description: ……

Please turn the page for the remaining data collection sections

**Please provide any additional information about the event?**<br><br>*For example:*<br>** Drug initiated/removed/changed shortly before harm was occurred*<br>** Removal of the drug improve the patient's condition*	……………………………………………………………………<br>……………………………………………………………………<br>……………………………………………………………………<br>……………………………………………………………………<br>……………………………………………………………………<br>……………………………………………………………………

**What was the severity of the ME** *(Please check only one of the NCC MERP index provided below)*		
☐ **A. Circumstances or events that have the capacity to cause error**<br>Description:……………………………<br>…………………………………………<br>…………………………………………	☐ **B. An error occurred but the error did not reach the patient (An "error of omission" does reach the patient)**<br>Description:…………………………………<br>………………………………………………<br>………………………………………………	☐ **C. An error occurred that reached the patient but did not cause patient harm**<br>Description:…………………………<br>…………………………………………<br>…………………………………………
☐ **D. An error occurred that reached the patient and required monitoring to confirm that it resulted in no harm to the patient and/or required intervention to preclude harm**<br>Description:……………………………<br>…………………………………………<br>…………………………………………	☐ **E. An error occurred that resulted in the need for treatment or intervention and caused temporary patient harm.**<br>Description:…………………………………<br>………………………………………………<br>………………………………………………	☐ **F. An error occurred that resulted in initial or prolonged hospitalisation and caused temporary harm.**<br>Description:……………………………<br>…………………………………………<br>…………………………………………
☐ **G. An error occurred that resulted in permanent patient harm.**<br>Description:……………………………<br>…………………………………………<br>…………………………………………	☐ **H. An error occurred that resulted in near-death event** (e.g. anaphylaxis, cardiac arrest)<br>Description:…………………………………..<br>………………………………………………..<br>………………………………………………..	☐ **I. An error occurred that resulted in patient death.**<br>Description:……………………………<br>…………………………………………<br>…………………………………………<br>…………………………………………

End of ME information collection form
